# Prevalence and Factors Associated with Self-Reported Vision Disability among the Elderly in Malaysia: Findings from National Health and Morbidity Survey (NHMS) 2018

**DOI:** 10.1155/2021/7564827

**Published:** 2021-05-03

**Authors:** Muhammad Solihin Rezali, Nor' Ain Ab Wahab, Norhafizah Sahril, Muhd Hafizuddin Taufik Ramli, Nik Adilah Shahein, Ying Ying Chan, Nur Liana Ab Majid, Mohd Hasnan Ahmad, Mohd Shaiful Azlan Kassim

**Affiliations:** Institute for Public Health, National Institutes of Health, Ministry of Health Malaysia, Shah Alam, Selangor, Malaysia

## Abstract

**Introduction:**

Disability has adverse effects on health, wellbeing, and life quality. Vision disorder is one of the top-ranked causes of disability in the elderly population. This study aims to determine the prevalence and factors associated with vision disability among the elderly in Malaysia. *Methodology*. Data collection from National Health and Morbidity Survey (NHMS) 2018 was obtained. This survey focused on elderly health by using two-stage stratified cluster sampling design. The Washington Group Extended Question Set on Functioning (WG ES-F) was used to determine the vision disability. The data were analyzed using SPSS version 21.0 utilizing a complex sample design with multivariable logistic regression analysis to determine the prevalence and associated factors to vision disability.

**Results:**

A total of 3,977 elderly completed the vision disability questionnaire. The overall prevalence of vision disability among those who were 60 years old and above was 4.5%. Multiple logistic regression revealed that no formal education (AOR: 6.69, 95% CI: 1.52, 29.49), only primary education (AOR: 4.26, 95% CI: 1.01, 18.03), unemployed/retiree or homemaker (AOR: 3.25, 95% CI: 1.79, 5.89), hypertension (AOR: 1.45, 95% CI: 1.00, 2.09), and malnourished elderly (AOR: 2.84, 95% CI: 1.76, 461) had higher odds for having vision disability.

**Conclusion:**

The findings suggest that a low education level, unemployment, hypertension, and malnourishment are significant risk factors for VD among Malaysia's elderly. Strengthening awareness campaigns to increase VD awareness and provide high-quality rehabilitation services must target specific groups, such as the elderly with a low level of education and the unemployed. Empower primary healthcare providers with the knowledge and skills necessary to improve the quality of eye care delivery and expand eye screening in settling VD issues nationally.

## 1. Introduction

The population of the world is aging. Almost every country in the world has an increasing number of elderly in its population [1]. The World Health Organization (WHO) projects that the proportion of the global population over 60 years of age will increase almost twice between 2015 and 2050, from 12% to 22% [2]. Malaysia is on the edge of the aging population by 2020, whereby the percentage of the population aged 65 years old and above reached 7.2% of the total population. This percentage is expected to double in 2040 to 14.5% (6 million) of the total population [3]. Given the increasing global population, all nations will face significant challenges in ensuring that they are ready to serve the aging population with their health and social systems.

Disabilities are a broad category, including impairments, activity restrictions, and engagement limitations [4]. Sensory organ disability (vision and hearing) is the top-ranked cause of disability in the elderly population [5]. Vision disability (VD) was defined as subjects with visual impairment or blindness. The World Health Organization categorized visual impairment into two groups, distance and near presenting vision impairment. Distance vision impairment is defined as presenting visual acuity that is worse than 6/12 to better than 6/60 in the better-seeing eye, while blindness is when presenting visual acuity is worse than 3/60. Near vision impairment defines visual acuity worse than N6 with existing correction [6]. Up to 285 million people worldwide are estimated to have some VD, and 39 million people are blind, 82% of whom are 50 years of age and above [7].

People with disabilities worldwide face many barriers that impede their involvement in all life facets, including attitudinal, environmental, or institutional barriers. Many older people with disabilities face more age barriers in society and are among the most negatively affected. VD has adversely affected physical and mental health. Individuals who are vision-disabled are more susceptible to depression, loneliness, and decreased physical activity [8, 9]. VD also affects productivity and increases the economic burden of a country. With a growing aging population and increased life expectancy of the worldwide population, the burden of vision disability is expected to increase.

The government has made much more effort to tackle the challenges faced by the aging society of Malaysia, particularly for the elderly with disabilities. [10]. Data on vision disability and its associated factors are essential for policymakers and stakeholders in developing policies, programs, and strategies for minimizing Malaysia's vision disability. Thus, this study was conducted to determine the prevalence of self-reported VD and its associated factors among the elderly in Malaysia.

## 2. Methodology

### 2.1. Sampling Design and Study Population

The NHMS 2018 was a nationally representative health survey for older populations aged 50 years and above in Malaysia. It was a cross-sectional survey that employed a two-stage stratified cluster sampling design. The first stage sampling unit was the enumeration block (EB) and the second stage sampling unit was the living quarters (LQ). These EBs constituted the sampling frame for the NHMS 2018. The selection of EBs was carried out independently from all 13 states and 3 federal territories in Malaysia (as primary stratum) and within urban or rural areas (as secondary stratum). The Department of Statistics Malaysia conducted selection of samples, with 60 enumeration blocks (EBs) in the urban areas and 50 EBs in the rural areas. The allocation of samples to the state and strata was done proportionally to the population size. Eligible respondents were those staying in the selected LQ for the past 2 weeks, aged more than 50 years, and able to communicate on their own or through a proxy. Respondents aged 60 years and above were included for purpose of this report [11].

### 2.2. Data Collection

From August until September 2018, field data collection was performed. Before conducting the survey, a bilingual (Malay and English) standardized questionnaire was developed, pretested, and piloted. Trained researchers interviewed respondents face to face using mobile devices. For those who had communication difficulties such as poststroke, cognitive disability, or speech disorders, including language barriers, proxies were used. Typically, the proxy was the family member who knew the respondent best. Before interviews, informed consent was obtained from all eligible respondents. Verbal consent was acquired from illiterate participants with thumbprints on the consent forms, with a literate individual as the witness. The individual response rate of this study was 95.8%.

### 2.3. Measures

#### 2.3.1. Dependent Variable

Vision disability as a dependent variable was assessed via the Washington Group Extended Questions Set on Functioning (WG ES-F). The WG ES-F questionnaire was created based on the International Classification of Functioning, Disability, and Health (ICF) framework by the WHO for producing internationally comparable data [4]. It is a self-rated assessment that focuses on functional limitations rather than impairments. The utilized questionnaire consists of 4 questions, including assistive devices/aids (glasses or contact lenses) that recognize persons who report seeing difficulties and identify those who have near vision difficulty, far vision difficulty, or both within this category. These four questions are as follows:

- Do you wear glasses/contact lenses? (yes/no)
- Do you have difficulty seeing (even when wearing your glasses/contact lenses)? (no difficulty/some difficulty/a lot of difficulties/cannot see at all)
- Do you have difficulty clearly seeing someone's face at a distance of 6 meters or 20 feet (even when wearing your glasses/contact lenses)? (no difficulty/some difficulty/a lot of difficulties/cannot see at all)
- Do you have difficulty clearly seeing the picture on a coin (even when wearing your glasses/contact lenses)? (no difficulty/some difficulty/a lot of difficulties/cannot see at all)

The levels of difficulties are grouped into four discrete categories: “no difficulty,” “some difficulty,” a lot of difficulties,” and “cannot see at all.” A dichotomous group was based on positive or negative responses. A positive response characterized as answering either “a lot of difficulties” or “cannot see at all” to any of the questions was classified as having vision disability and vice versa [12].

#### 2.3.2. Independent Variables

Independent variables included in this study were sociodemographic, non-communicable diseases, and nutritional status variables. Sociodemographic variables included age (60–69, 70–79, and ≥ 80 years), gender (male and female), strata area (urban and rural), ethnicity (Malay, Chinese, Indian, other Bumiputera, and others), education (no formal education, primary, secondary, and tertiary education), marital status (married and never married/separated/divorced/widowed), and employment status (employed and unemployed/retired/homemaker).

Presence of medically diagnosed diabetes and hypertension was defined as the participant self-reported having been told by a medical doctor that he or she had that specific condition. Current smoker was defined as currently using any smoked tobacco products (manufactured cigarettes, hand-rolled cigarettes, kretek, cigars, shisha, bidis, or tobacco pipes) at the survey time.

Nutritional status variables were assessed using a validated Malay version of the Mini Nutrition Assessment-Short Form (MNA-SF). The MNA-SF test comprises anthropometric measurements (body mass index, weight loss), global assessment (mobility), dietary questionnaire, and subjective assessment (food intake, neuropsychological problems, acute disease). Total scores of MNA-SF ranged from 0 to 14. The range of total score below 8, 8 to 11, and above 11 indicates malnutrition, risk of malnutrition, and normal, respectively [11].

### 2.4. Statistical Analysis

The data set was clarified and edited for inconsistencies. Data was analyzed using Statistical Package for the Social Sciences (SPSS) version 21.0, utilizing a complex survey design. Prevalence of overall VD among the elderly 60 years old and above in Malaysia and its predictors by sociodemographic profiles, noncommunicable diseases, and nutritional status were estimated using descriptive and multivariable logistic analyses. The final model was created, including all predictors significantly associated at a level of *p* value <0.05.

### 2.5. Ethical Approval

This research was approved by the National Medical Research Register of Malaysia (NMRR-17-2655–39047) and Medical Research and Ethics Committee of the Ministry of Health Malaysia (KKM.NIHSEC.P18-49(6)).

## 3. Results

Out of successfully interviewed 5,017 living quarters, 3,977 eligible respondents were aged 60 years old and above in this study.

Table 1 shows distribution of response by dependent variables (WG ES-F) and 63.5% of total respondents wore a corrective device (glasses/contact lenses).  Table 2 shows sociodemographic characteristics of the respondents 60 years and above in NHMS 2018. The mean age was 68.3 years. Rural dwellers were slightly more than urban dwellers. By education level, half of the respondents were primary school educated, and one-fifth had no formal education. Chronic illnesses such as diabetes mellitus and hypertension took up to 25.6% and 51.1%, respectively. One-third of the elderly in Malaysia had malnutrition.

From the study, the overall prevalence of VD among 60 years and above was 4.5% (95% CI: 3.42, 5.90). The prevalence of VD was higher among the older age group, among rural dwellers, among lower educational status, and among unemployed/retired/homemakers as shown in Table 3.

As shown in Table 3, complex sample multiple logistic regression analysis revealed that the elderly with no formal education, primary school education, unemployed/retiree or homemaker, having hypertension, and being malnourished were significantly associated with vision disability.

## 4. Discussion

Based on our survey findings, the prevalence of the elderly aged 60 and above who had VD was 4.5%. This finding was comparable with our previous study in the National Health and Morbidity Survey (NHMS) 2015, which reported that prevalence of VD among those 60 years and above was 4.3% (95% CI 3.3, 5.5) [13]. In other studies, utilizing similar questionnaires in Malawi and India, the prevalence of self-reported VD was 4.2% and 1.0%, respectively [14, 15]. A widely cited report by Bourne et al. found that the global age-standardized, all-age prevalence of moderate and severe vision impairment was 2.90% (95% CI 1.31, 4.80), and the majority of affected individuals resided in South Asia, East Asia, and Southeast Asia [16]. Due to that, we are interested in reporting the prevalence of vision disability among the elderly and the risk factors in Malaysia.

Globally, VD's leading causes among the elderly, most of which are avoidable and treatable, are uncorrected refractive error, cataract, and age macular degeneration [16]. The WHO estimated 123.7 million population affected by unaddressed refractive error [6]. Vision2020 by the WHO listed refractive errors as one of the top five priorities for the Global Initiative for the Elimination of Avoidable Blindness [17]. A nationwide population-based study done in Malaysia, National Eye Survey (NESII), estimated the prevalence and causes of VD among the elderly in Malaysia. From the study, uncorrected refractive error (14.4%) came as second place for the commonest causes of VD after untreated cataract (68.0%) and diabetic retinopathy (6.1%) [18]. The high prevalence of the elderly with glasses or contact lenses warrants promoting eye screenings and provision of affordable correction devices (glasses/contact lenses) among the population by the Malaysian government, in particular the older generation.

This study identified the elderly population subgroups at risk of having VD, particularly those with low educational levels and unemployed elderly. These groups were more prone to having VD compared to others. The elderly with no formal education or primary school education had six times and four times risk to have VD from the multivariate analysis. The relationship between educational level and VD is also seen in various studies [19–21]. Educated people were less likely to have VD. One potential explanation may be that people with a lower level of education may have limited knowledge of their health conditions. When referred to tertiary eye care for further treatment, they were less likely to undergo regular eye health screening and more likely to have lower compliance [22]. Education enhances personal understanding and awareness and promotes access to information deemed essential for self-wellness [14, 21–23].

The unemployed elderly were three times at risk of having VD. The effect of unemployment and vision disability was found in multiple studies [24, 25]. A visually disabled person was expected to have low chances of getting employment [14], and they were more likely to earn a lower income than a nondisabled person even though they were working [26]. A study from Mojon-Azzi et al. reported that people with self-reported low general eye levels were considerably less pleased with their jobs, felt less freedom of choice, had fewer opportunities in challenging circumstances to learn new skills, and less acceptance for work, and had an insufficient salary [27]. These would hinder them from getting proper healthcare services. It is vital to promote blindness prevention, quality eye care, and rehabilitation service provision to target groups such as the elderly with a lower education level and the unemployed.

As per comorbid status, hypertension was a prominent risk factor of VD among the elderly in Malaysia. Poorly controlled hypertension can cause target-organ damage (TOD). One of the organs is the eye; high blood pressure can affect and damage the retinal vessels, leading to hypertensive retinopathy condition [28]. Three forms of ocular damage are caused by this affecting the eye: choroidopathy, retinopathy, and optic neuropathy [29]. In a cross-sectional study in Central Africa, 83.6% of the total hypertensive patients recorded hypertensive retinopathy occurrence [30]. Another study reported that people with hypertension for more than ten years showed a high prevalence of visual disability [31].

According to WHO, malnutrition in the elderly varies from 1.3% to 47.8% [32]. In Malaysia, the prevalence of malnutrition among the elderly was 7.3% (CI: 6.0–8.9) [11]. Based on nutritional status, the elderly with malnutrition showed almost three times higher VD risk than those with normal nutritional status. Singapore Longitudinal Ageing Studies indicated that VD is twice associated with elderly with malnutrition [33]. A systematic analysis by Jones et al. found that VD has a substantial effect on the nutritional status by making it difficult for visually disabled people to cope with shopping, meal preparation, and restaurant use [34]. There is, however, evidence from RCTs that prophylactic antioxidant supplementation with vitamins or minerals does not prevent the production of AMD or cataracts [35, 36]. Although nutrients play a vague role in preventing or slowing age-related eye disease, a balanced diet is essential for healthy aging. There is well-documented evidence of the role of optimum nutrients in maintaining health and preventing chronic diseases (cardiovascular disease, diabetes mellitus, hypertension) and some cancers [37–39]. Future research is needed to improve our understanding of nutritional strategies' role in improving eye health [40].

The 73rd World Health Assembly adopted resolution WHA73.4 entitled “Integrated people-centred eye care, including preventable vision impairment and blindness.” To accomplish this, the WHO identified three enabling factors: improving eye care delivery, especially through primary healthcare; improving eye care information systems; and strengthening the eye care workforce [41]. Malaysia, being one of the member states, also endorsed and signed this resolution. Vision disability among the elderly continues to be a significant health issue in our country, urging for more reliable data and association factors on vision disability to develop policies, programs, and strategies in Malaysia. For instance, Malaysia's government introduced PeKa B40, a federal program to assist Malaysian citizens in the bottom 40% household income range [42]. One of the health benefits is medical equipment aid; a maximum of RM20,000 is allocated for patients seeking treatment at government hospitals. Among the equipment given are intraocular lens, stent for heart diseases, artificial joints, hearing aid, wheelchairs, and oxygen concentrator. Patients receiving assistance from the Social Welfare Department (SWD) have been exempted from making any payment to undergo cataract surgery. The government has also organised numerous campaigns and activities to address VD, including World Sight Day, Glaucoma Awareness Day, Diabetic Retinopathy Screening, Cataract Finder Program, and routine eye screening in primary healthcare clinics.

### 4.1. Strengths and Limitations

This study has many strengths and limitations. The strength of this study comes from the fact that a large national representative sample of the elderly was used which contributes to the reliability and validity of the data. The use of an internationally comparable validated tool in this survey also enables international comparisons. There may, however, be some restrictions. This is a cross-sectional study that defines correlation but not cause. Furthermore, we have not been able to differentiate between individuals with pre-existing disabilities until they have entered the elderly group and others who have become disabled during their elderly years. Another downside to this survey is the use of self-reported functional limitation without clinical validation.

## 5. Conclusion

This survey offers comprehensive and globally comparable data on vision disability among the elderly in Malaysia. In conclusion, our findings suggested that a low education level, unemployment, hypertension, and malnourishment are significant risk factors for VD among Malaysia's elderly. Improving eye care information systems for the general population, not just the elderly, is essential in addressing the issues. Advocate for eye healthcare through initiatives and campaigns, as well as through social media, in order to raise awareness of eye healthcare among the general public. A high-quality rehabilitation program will significantly increase the quality of life for a disabled person. Primary healthcare providers should be empowered with the knowledge and skills to improve eye care delivery. Additionally, nationwide eye screening focusing on susceptible groups is beneficial in reducing the burden of disability causes.

## Figures and Tables

**Table 1:** Distribution of dependent variables using the Washington Group Extended Question Set on Functioning (WG ES-F).

Domains		Prevalence (%) (95% CI)
Respondents wear glasses/lenses	Yes	65.3 (62.8, 67.8)
	No	34.7 (32.2, 37.2)

Respondents with difficulty seeing (even when wearing glasses/lenses)	Have difficulty	2.8 (2.2, 3.7)
	No difficulty	97.2 (96.3, 97.8)

Respondents with difficulty clearly seeing someone's face across a room (even when wearing glasses/lenses)	Have difficulty	3.4 (2.6, 4.5)
	No difficulty	96.6 (95.5, 97.4)

Respondents with difficulty clearly seeing the picture on a coin (even when wearing glasses/lenses)	Have difficulty	2.8 (2.1, 3.8)
	No difficulty	97.2 (96.2, 97.9)

**Table 2:** Sociodemographic characteristics of the elderly (60 years and above), NHMS 2018 (*n* = 3977).

Sociodemographic characteristics	Unweighted count (*n*)	Percentage (%)
*Age* (mean±SD)	68.30	(6.95)

*Gender*		
Male	1872	48.9
Female	2105	51.1

*Residence*		
Urban	1689	42.5
Rural	2288	57.5

*Ethnic group*		
Malay	2581	64.9
Chinese	710	17.9
Indian	126	3.2
Other Bumiputera	446	11.2
Others	114	2.9

*Education level*		
No formal education	806	20.3
Primary school education	1939	48.8
Secondary school education	967	24.3
Tertiary education	265	6.7

*Marital status*		
Married	2624	66.0
Never married/separated/divorced/widowed	1350	34.0

*Occupation status*		
Employed	1050	26.4
Unemployed/retiree/homemaker	2927	73.6

Diabetes mellitus	1018	25.6
Hypertension	2027	51.1
Current smoker	622	15.7
Malnutrition	1419	35.7

**Table 3:** Prevalence and associated factors of vision disability among the elderly (60 years and above) in Malaysia.

Variables	Prevalence (95% CI)	Crude OR (95% CI)	Adjusted OR^a^ (95% CI)
Overall	4.5 (3.42, 5.90)	—	—

*Age group*			
60–69	3.3 (2.31, 4.81)	*R*	—
70–79	6.4 (4.67, 8.63)	1.96 (1.23, 3.02)	1.19 (0.81, 1.77)
80+	8.5 (5.62, 12.75)	2.69 (1.68, 4.32)	1.09 (0.67, 1.77)

*Gender*			
Male	4.4 (3.11, 6.22)	*R*	—
Female	4.6 (3.35, 6.35)	1.10 (0.69, 1.58)	0.65 (0.39, 1.07)

*Residence*			
Urban	3.8 (2.57, 5.62)	*R*	—
Rural	6.5 (4.80, 8.61)	1.74 (1.04, 2.91)	1.18 (0.60, 2.31)

*Ethnic group*			
Malay	4.4 (3.16, 6.02)	*R*	—
Chinese	4.4 (2.58, 7.29)	0.99 (0.54, 1.84)	1.30 (0.65, 2.60)
Indian	3.1 (1.48, 6.37)	0.69 (0.30, 3.47)	0.68 (0.27, 1.69)
Other Bumiputera	8.1 (4.92, 12.96)	1.92 (1.06, 3.47)	1.36 (0.67, 2.73)
Others	1.8 (0.55, 5.56)	0.39 (0.11, 1.35)	0.34 (0.10, 1.14)

*Education level*			
No formal education	9.4 (6.84, 12.71)	9.21 (3.12, 27.19)	6.69^*∗*^ (1.52, 29.49)
Primary school education	5.3 (3.97, 7.13)	5.01 (1.64, 15.34)	4.26^*∗*^ (1.01, 18.03)
Secondary school education	2.3 (1.09, 4.59)	2.05 (0.62, 6.82)	2.32 (0.47, 11.54)
Tertiary education	1.1 (0.37, 3.30)	*R*	—

*Marital status*			
Married	3.9 (2.89, 5.31)	*R*	—
Never married/separated/divorced/widowed	5.8 (4.14, 8.04)	1.51 (1.05, 2.17)	0.98 (0.65, 1.47)

*Occupation status*			
Employed	1.7 (0.96, 3.02)	*R*	—
Unemployed/retiree/homemaker	5.4 (4.15, 7.05)	3.31 (1.99, 5.48)	3.25^*∗*^ (1.79, 5.89)

*Diabetes mellitus*			
Yes	5.3 (3.66, 7.56)	1.26 (0.81, 1.96)	1.20 (0.72, 1.99)
No	4.2 (3.08, 5.81)	*R*	—

*Hypertension*			
Yes	5.6 (4.05, 7.57)	1.64 (1.22, 2.22)	1.45^*∗*^ (1.00, 2.09)
No	3.5 (2.61, 4.55)	*R*	—

*Current smoker*			
Yes	6.1 (3.42, 10.56)	1.45 (0.74, 2.81)	1.36 (0.64, 2.92)
No	4.3 (3.17, 5.75)	*R*	—

*Nutritional status*			
Normal	2.6 (1.69, 4.02)	*R*	—
Malnutrition	8.8 (6.82, 11.32)	3.61 (2.28, 5.71)	2.84^*∗*^ (1.75, 4.61)

CI: confidence interval; OR: odds ratio. ^a^Adjusted for age group, gender, residence, ethnic group, educational level, marital status, occupation status, diabetes mellitus, hypertension, current smoker, and nutritional status. ^*∗*^Significant <0.05 for logistic regression.

## Data Availability

The data used to support the findings of this study are available from the corresponding author upon reasonable request.
